# Grossesse molaire partielle avec fœtus diploïde vivant: à propos d’un cas et revue de la littérature

**DOI:** 10.11604/pamj.2020.36.90.23592

**Published:** 2020-06-15

**Authors:** Dhekra Toumi, Ahmed Hajji, Wael Mbarki, Soumaya Kraiem, Haifa Bouchahda

**Affiliations:** 1 Service de Gynécologie-Obstétrique, CHU Tahar Sfar, Mahdia,; 2 Service de Gynécologie-Obstétrique, CHU Fattouma Bourguiba, Monastir, Tunisie

**Keywords:** Môle partielle, caryotype diploïde, fœtus vivant, Partial mole, diploid karyotype, liveborn fetus

## Abstract

La môle hydatiforme partielle (MHP) fait partie des maladies trophoblastiques gestationnelles. Appelée également môle embryonnée, il s’agit d’un œuf humain pathologique comportant des villosités en transformation vésiculaire, mais conservant une forme placentaire reconnaissable et une cavité amniotique avec un fœtus. La circonstance diagnostique la plus commune étant le tableau d’avortement spontané au premier trimestre. Rarement les môles partielles persistent au-delà du premier trimestre et sont alors source de complications maternelles et fœtales et de confusion diagnostique. L’origine génétique des MHP correspond à une conception triploïde avec un lot chromosomique supplémentaire d’origine paternelle. La coexistence d’un fœtus de caryotype normal avec une MHP est une situation exceptionnelle. Nous rapportons un cas rare de grossesse molaire partielle avec fœtus vivant diploïde à 27 semaines d’aménorrhée (SA) chez une femme âgée de 36 ans dont le diagnostic est porté à l’occasion d’une menace d’accouchement prématurée associée à un placenta prævia.

## Abstract (en)

Partial hydatiform mole (MHP) represents a spectrum of trophoblastic-related disorders occurring during pregnancy. Also known as embryonal mole, it is characterized by a recognizable ovum abnormality with vesicular transformation of villi but with recognizable placental appearance and amniotic cavity containing the fetus. First-trimester spontaneous abortion most commonly suggests the diagnosis. Partial moles rarely persist beyond the first trimester and are then a cause of maternal and fetal complications and diagnostic confusion. MHP of genetic origin is triploid with extra chromosome of paternal origin. The coexistence of normal fetal karyotype and MHP is exceptional. We report a rare case of partial molar pregnancy with liveborn diploid fetus in a 36-year-old woman diagnosed with threat of premature labour associated with placenta previa at 27 weeks of amenorrhea (WA).

## Introduction

La môle hydatiforme partielle (MHP) fait partie des maladies trophoblastiques gestationnelles. Elle est plus fréquente que la môle hydatiforme complète avec une incidence de 3 pour 1000 grossesses [1]. Le seul facteur de risque qui a été clairement identifié est l’âge maternel [2, 3]. Elle se caractérise par une dégénérescence hydropique focale du placenta associant un sac gestationnel reconnaissable et un fœtus avec une sécrétion excessive de l’hormone choriogonadotrophine (HCG). L’origine génétique des MHP correspond à une conception triploïde avec un lot chromosomique supplémentaire d’origine paternelle [4]. L’association d’un fœtus vivant avec un caryotype normal est une situation très rare survenant dans 0,005 à 0,01% de toutes les grossesses et le diagnostic dans ce cas est souvent difficile, surtout en l’absence de signes cliniques révélateurs [4, 5]. Nous rapportons un cas rare de grossesse molaire partielle diagnostiquée tardivement à 27 semaines d’aménorrhée avec issu d’un fœtus de caryotype normal.

## Patient et observation

**Figure 1:**
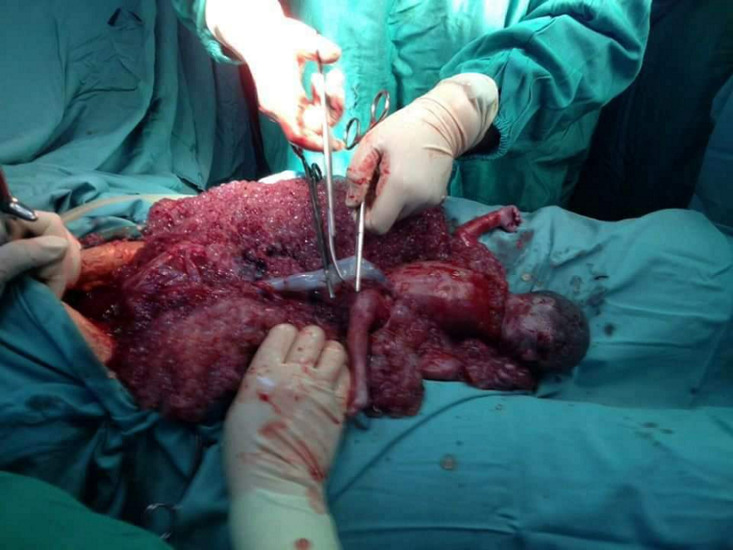
vue per opératoire mettant en évidence l’aspect vésiculaire du placenta et le morphotype normal du nouveau-né

Notre patiente âgée de 36 ans, ayant 2 enfants vivants, s’est présentée aux urgences pour des douleurs pelviennes aiguës associées à des métrorragies sur terme de 27SA + 3 jours. L’interrogatoire n’a pas relevé d’antécédents particuliers, la grossesse était non suivie, aucune consultation prénatale ni échographie n’ont été réalisées. L’examen clinique a objectivé une tension artérielle à 170/100 mmHg, la protéinurie à la bandelette était positive à quatre croix. La hauteur utérine était à 29 cm, avec des contractions utérines (CU) rapprochées. Une échographie réalisée en urgence a objectivé une grossesse évolutive en présentation transverse avec une biométrie de 24-25 SA et un placenta hypertrophié bas inséré recouvrant le col d’aspect vésiculaire. Un toucher vaginal réalisé avec prudence après conditionnement au bloc opératoire a retrouvé un col dilaté à 8 cm. Le bilan a montré une hémoglobine à 4,2 g/L, le dosage de la βHCG revenu à 96000 UI/L. Une césarienne en urgence a été indiquée pour sauvetage maternel. En per opératoire, l’utérus était augmenté de taille d’aspect gravide normal. Une incision segmentaire était pratiquée avec extraction transplacentaire d’un fœtus en état de mort apparente de morphologie normale, de sexe féminin avec un poids de naissance à 700 g. Le fœtus est transféré en unité de soins intensifs après intubation. Le placenta était d’aspect vésiculaire pesant 3060 g (Figure 1). Le caryotype du nouveau-né revenu normal (46XX). L’examen anatomopathologique du placenta a confirmé le diagnostic d’une MHP. L’étude chromosomique du placenta a montré un caryotype triploïde 69 XXX. En post opératoire ; la parturiente n’a pas présenté de complications et le taux de βHCG a chuté rapidement. Le nouveau-né est décédé à J 2 de vie par détresse respiratoire liée à la grande prématurité.

## Discussion

Les grossesses molaires sont classées comme des conceptions non viables [6]. Il s’agit d’une anomalie de la conception, qui se manifeste par une croissance excessive du placenta et l’absence d’un développement fœtal normal. Ces anomalies dérivent généralement de la fécondation dispersée d'un ovocyte haploïde normal et produit un ensemble triploïde de chromosomes [7]. En présence d’une triploïdie, le fœtus ne peut pas survivre après la naissance en raison des malformations multiples et du retard de croissance intra-utérin sévère secondaire à la circulation placentaire affectée.

La coexistence d’un fœtus diploïde avec une MHP est une situation extrêmement rare au cours de laquelle le fœtus peut survivre à terme [5, 8]. Devant cette association, le diagnostic différentiel principal étant une grossesse gémellaire avec un fœtus diploïde et un placenta normal et un deuxième placenta en môle complète où la première évaluation s’intéresse à la recherche d’un placenta séparé normal [9]. La dysplasie mésenchymateuse placentaire est une lésion vasculaire placentaire qui constitue également un diagnostic différentiel rare avec la MHP qu’il ne faut pas méconnaitre [10].

Le diagnostic précoce d’une MHP conduit dans la majorité des cas à une interruption de la grossesse d’une part du fait de la fréquence des triploïdies et d’autre part du fait du risque maternel et la possibilité d’évolution vers la maladie trophoblastique persistante ultérieurement [11, 12]. Une surveillance étroite de la mère et du fœtus peut aider à atteindre un résultat favorable, et l’évacuation de la grossesse n’est requise que dans les cas des anomalies fœtales ou une détérioration de l'état maternel [13]. Plusieurs facteurs peuvent affecter l'issue du fœtus en cas de grossesse molaire partielle. Ceux-ci incluent le caryotype fœtal, la taille du placenta anormal, la vitesse de la dégénérescence molaire et l'apparition d’une anémie fœtale ou autres complications obstétricales telle que la prématurité [14].

## Conclusion

Malgré la connaissance parfaite des mécanismes physiopathologiques des anomalies chromosomiques lors des grossesses môlaires, la forme partielle avec fœtus diploïde reste une pathologie qui prête à confusion du fait de l’absence souvent d’arguments cliniques en faveur du diagnostic. Une grande vigilance est nécessaire afin de les suspecter précocement pour réaliser un diagnostic fiable permettant une prise en charge optimale.
